# Split it up and see: using proxies to highlight divergent inter-populational performances in aquaculture standardised conditions

**DOI:** 10.1186/s12862-021-01937-z

**Published:** 2021-11-22

**Authors:** Lola Toomey, Simon Dellicour, Andrzej Kapusta, Daniel Żarski, Frederik Buhrke, Sylvain Milla, Pascal Fontaine, Thomas Lecocq

**Affiliations:** 1 University of Lorraine, INRAE, URAFPA, 54000 Nancy, France; 2 Spatial Epidemiology Lab (SpELL), Université Libre de Bruxelles, 50 av. FD Roosevelt, CP160/12, 1050 Brussels, Belgium; 3 Department of Microbiology, Immunology and Transplantation, Rega Institute, Laboratory for Clinical and Epidemiological Virology, KU Leuven - University of Leuven, Leuven, Belgium; 4 Department of Ichthyology, Hydrobiology and Aquatic Ecology, Inland Fisheries Institute, ul. Oczapowskiego 10, 10-719 Olsztyn, Poland; 5 Department of Gamete and Embryo Biology, Institute of Animal Reproduction and Food Research, Polish Academy of Sciences, ul. Tuwima 10, 10-748 Olsztyn, Poland; 6 Mecklenburg-Vorpommern Research Centre for Agriculture and Fisheries, Institute of Fisheries, Research Station Aquaculture, 18375 Born, Germany

**Keywords:** Proxy, Distance, Aquaculture, Domestication, *Perca fluviatilis*

## Abstract

**Background:**

Considering wild inter-populational phenotypic differentiation can facilitate domestication and subsequent production of new species. However, comparing all populations across a species range to identify those exhibiting suitable key traits for aquaculture (KTA; i.e. important for domestication and subsequent production) expressions is not feasible. Therefore, proxies highlighting inter-populational divergences in KTA are needed. The use of such proxies would allow to identify, prior to bioassays, the wild population pairs which are likely to present differentiations in KTA expressions in aquaculture conditions. Here, we assessed the relevance of three alternative proxies: (i) genetic distance, (ii) habitat divergence, and (iii) geographic/hydrologic distances. We performed this evaluation on seven allopatric populations of *Perca fluviatilis* for which divergences in KTA had already been shown.

**Results:**

We showed differences in the correlation degree between the alternative proxy-based and KTA-based distance matrices, with the genetic proxy being correlated to the highest number of KTA. However, no proxy was correlated to all inter-populational divergences in KTA.

**Conclusion:**

For future domestication trials, we suggest using a multi-proxy assessment along with a prioritisation strategy to identify population pairs which are of interest for further evaluation in bioassays.

**Supplementary Information:**

The online version contains supplementary material available at 10.1186/s12862-021-01937-z.

## Background

Inter-populational differentiation is the divergence between allopatric, peripatric, or parapatric conspecific populations (e.g. in fishes [1] and insects [2]). It results from specific demographic history, limited gene flow, random genetic drift, and/or local adaptations [3, 4]. Often considered in evolutionary and conservation biology [5], analysing and exploiting inter-populational differentiation is also of crucial interest for agriculture development. Indeed, populations undergoing inter-populational differentiation process can also present divergent expressions of key traits, which are essential phenotypic traits for domestication and subsequent production (see examples of key traits for fishes in [6, 7]). Therefore, considering population specificities could allow identifying population(s) which might facilitate farming of a new species [7–9]. Several success-stories have highlighted the interest of considering inter-populational specificities. For instance, the buff-tailed bumblebee (*Bombus terrestris*), which has been domesticated to act as pollinator in greenhouses, displays phenotypic differentiations between allopatric wild populations for traits impacting its ability to be industrially produced and to pollinate valuable crops [10]. At the beginning of its production, bumblebee breeders tried to rear several wild populations [10] and, eventually, identified one with superior characteristics, triggering a flourishing development of the bumblebee production industry [11]. Similarly, the development of the Atlantic salmon (*Salmo salar*) industry was facilitated by reaping the benefits from inter-populational differentiation. Initial comparisons of several key traits for fish farmers (e.g. growth rate, disease resistance) between wild populations allowed identifying those with higher performances which were then used as cornerstone fish stock of salmon production [12, 13]. Beside new domestication instances, wild intraspecific differentiation can also be used to enhance old-established species farming, because introduction of adaptive traits (i.e. genomic introgression) from wild populations can lead to phenotypic novelty in produced stocks [14].

Given the potential benefits of considering inter-populational phenotypic differentiation in domestication/farming programs, there is a premium on highlighting wild populations with divergences in key traits. This requires population assessments that can only be achieved through bioassays in which expressions of key traits are evaluated in a common experimental environment (i.e. common garden) close to future farming conditions. Similar assessments in natura would be doubtful because (i) some traits cannot be characterised during fieldworks due to technical reasons, and/or (ii) trait expression is the result of both genetic and environmental factors. The latter point is the main issue because phenotypic plasticity (sensu [15]) can shape observed divergences between wild populations. However, such divergences would be useless for domestication programs because they will not be (i) observed in ex-situ farming and (ii) conserved over generations in production environment. Only genetically based differentiations are of interest for domestication because production occurs in a system that is often quite different from the wild environment. The common-garden experiments are an efficient way to assess such differentiations since, through population comparison, it can highlight inter-populational divergences with a genetic basis [16]. However, such experiments are cumbersome and expensive procedures and thus all populations inhabiting the species distribution range cannot be pragmatically compared by this approach. This raises the need of developing a strategy to limit the number of populations that should be further investigated by common-garden experiments. Randomly selecting some populations cannot seen as a solution because populations potentially valuable for aquaculture could be missed out. Moreover, from a phenotypic perspective, populations are not all notably divergent from each other, making comprehensive assessments uselessly time/money consuming. Therefore, one solution relies on limiting the number of populations to be compared before performing bioassays by (i) gathering populations that are likely undifferentiated and (ii) highlighting populations/population groups that most likely display specificities or, at least, divergences in key traits.

Already available intraspecific taxonomic classifications like subspecies [17], historically widely defined (e.g. [18, 19]), could provide the needed population sorting. Although such classifications are not based directly on pieces of information about key traits (e.g. genes coding for key traits), their potential relevance was highlighted with the *B. terrestris* domestication in which inter-populational differentiation of key traits matches with the subspecies [11]. However, these taxonomic statuses have been fiercely criticised because (i) their relevance for systematics and (ii) criteria that should be considered for their definitions are doubtful [19, 20]. This has triggered a trend to eliminate the trinomial designation in several species groups (e.g. [21, 22]). In conservation biology, evolutionarily significant units, management units, distinct population segments, or designatable units, have been proposed to complement existing taxonomy and/or to go beyond systematists’ feud [23–25]. However, their interpretations and definition criteria vary among species and scientists (e.g. [26, 27]). Overall, debates and lack of consensus in systematics, evolutionary biology, and biological conservation make a ready-to-use population sorting approach unavailable for species domestication/production programs. Moreover, such classifications can highlight populations that are diagnosably distinct from other conspecific population groups but cannot detect the clines of variation that could lead to inter-populational divergences for key trait without clear geographic boundaries (e.g. [1, 28]). Yet, such divergences could also be useful for domestication programs. Therefore, there is a need to establish which approach(es) could be used to efficiently highlight populations that most likely display divergences in key trait expressions prior to their assessment by bioassays. This would ultimately allow selecting wild populations which need to be evaluated in farming conditions to determine which ones are the most interesting for further domestication and production.

One potential approach can be based on proxies which (i) are correlated with key traits and (ii) can be easily studied at large-scale. It is obvious that a good proxy should consist of using differentiation in genes under selection and coding for key traits. However, related methodologies, whether quantitative trait locus mapping or genome-wide association studies, are still underway, present some intrinsic limitations (e.g. explaining only a part of the phenotypic variance, cost and time consuming, knowledge of genome features), and/or are so far only developed on a limited number of species [29–32]. Therefore, alternative pragmatic proxies of phenotypic differentiation are needed.

A first alternative proxy consists of using neutral genes (i.e. locus that does not influence fitness). Indeed, divergences in such genes is widely used to highlight population groups, connected by a low or null gene flow, which underwent divergent demographic histories [4]. Considering previous observations (e.g. [33, 34]), one could expect that such groups could have also acquired some phenotypic specificities, including in key traits for production. Indeed, genetic differentiation in neutral markers has previously been suggested as, at least partially, indicative of differentiation in genes coding for quantitative loci ([35], but see opposite opinion in [36]). Therefore, neutral loci-based genetic distance could be a relevant proxy to discriminate populations that are most likely different in their key traits. A second alternative proxy is based on the degree of divergence of a phenotypic trait [18, 37] (e.g. behavioural, eco-chemical, or morphological features). Such features can be used to evaluate phenotypic distance that could be linked to genetic divergence [38, 39] and, therefore, potentially highlight divergences in key traits. A third alternative proxy relies on using the degree of habitat divergence to maximise the detection of population groups with different key trait expressions. Indeed, populations inhabiting different environments may be phenotypically divergent [40] because resulting distinct selective pressures act as driving forces for phenotypic differentiation, notably through genetic-based local adaptations [41, 42] that could impact key trait expression. Finally, a fourth alternative could be to consider spatial distance as a relevant proxy. Indeed, given the limited dispersal ability, individuals that are far apart tend to be genetically more divergent than individuals that are spatially close [43].

Overall, all these alternative strategies could provide a solution to highlight wild population groups with divergences in key traits for species production. However, the relevance of these alternative proxies is still unsettled because there is no comparison of their efficiency to discriminate populations which would likely express divergences in key trait expressions. Here, we aim to compare this relevance to highlight inter-populational divergences in the expression of key traits for aquaculture (KTA). We consider a part of the life cycle (larval stage) of seven allopatric populations of the European perch (*Perca fluviatilis*) as a test case. We evaluate a set of KTA considered as important for the larval production of this species [7, 33, 44]: (i) growth traits (here growth rate, final growth heterogeneity, initial and final lengths) [45], (ii) development (survival rate, deformity rate, swim bladder inflation rate) and nutrition (yolk sac volume) traits [46, 47], (iii) behavioural traits that are aggressiveness [48], inter-individual distances which reflect group structure [49], and swimming activity [50]. First, we evaluate these KTA expressions in the seven European perch populations. Second, we perform a retro-assessment of the three proxies on the same populations using the bioassays’ results. Finally, we compare the degrees of correlation between alternative proxy-based and KTA-based distance matrices to identify the most relevant proxies to highlight inter-populational divergences in KTA. Ultimately, we aim at providing guidelines for the application of proxies through a prioritisation procedure. This latter allows limiting the costly step of common garden experiment to the population pairs that are the most likely to display divergent KTA expressions.

## Results

### KTA evaluation in aquaculture standardised conditions

Inter-populational differences were assessed using Kruskal-Wallis or ANOVA F-tests (see Methods for details). There was no statistically significant difference between populations for aggressive rate (K = 8.85, df = 6, p-value = 0.18) and length heterogeneity (F_(6,14)_ = 0.86, p-value = 0.55).

A significant statistical differentiation between populations was found for survival rate (F_(6,14)_ = 9.45, p-value = 5.75 × 10^–4^), swim bladder inflation rate (F_(6,14)_ = 22.73, p-value = 6.79 × 10^–6^), deformity rate (F_(6,14)_ = 11.29, p-value = 1.12 × 10^–4^), specific growth rate (F_(6,14)_ = 8.64, p-value = 4.69 × 10^–4^), length at hatching (F_(6,14)_ = 17.33, p-value = 2.85 × 10^–5^), final length (F_(6,14)_ = 8.35, p-value = 5.58 × 10^–4^), yolk sac volume (F_(6,14)_ = 32.95, p-value = 1.77 × 10^–7^), activity (K = 20.24, df = 6, p-value = 0.003), and inter-individual distances (F_(6,56)_ = 5.59, p-value = 1.35 × 10^–4^). All inter-populational comparisons are available in Additional file 1: Fig. S1.

### Correlation degrees of the different proxies with the KTA-based matrices

The series of successive multivariate analyses using multi-regression on distance matrices coupled with commonality analyses (further referred as “MRDM-CA”) allowed identifying several suppressors (see Methods for methodology details). Therefore, some KTA are only correlated with a single proxy remaining after suppressors’ removal (Table 1). Overall, MRDM-CA results indicate that significant global MRDM R^2^ are relatively high (> 10%), and that all KTA are associated with a positive and significant correlation with a unique proxy-based matrix (when considering only proxies for which the commonality analysis [CA] unique contribution U > 5%), except for final length which is not correlated with any proxy (Table 1). The geographic distance proxy is not correlated with any KTA-based distance matrix while the genetic distance proxy is the one which is correlated to the highest number of KTA-based distances matrices. However, no proxy shows a systematic significant contribution with all KTA. Considering genetic, habitat, and hydrologic distance proxies, all KTA are correlated with at least one of the proxies, except for final length (Table 1). Regarding Mantel tests, results are available in Additional file 2: Fig S2 and are globally congruent with MRDM-CA analyses. Overall, for each KTA, the proxy which was the most correlated in the Mantel test is the one which was significant in the MRDM-CA analysis.

**Table 1:** Multi-regression (MRDM) and commonality analyses (CA) results after having successively removed all suppressors

KTA	R^2^ MRDM	CA				
		Proxy	r	β	U	C
Survival rate	0.432*	Genetic	0.505	0.874	–	–
Swim bladder inflation rate	0.399*	Genetic	0.631	0.638	–	–
Deformity rate	0.223*	Geographic	0.350	0.030	0	0.122
		Genetic	0.193	0.038	0.001	0.036
		Habitat	0.469	0.437	0.101	0.120
Specific growth rate	0.121*	Hydrologic	0.237	0.712	–	–
Initial length	0.224*	Genetic	0.472	0.500	–	–
Final length	0.023	Hydrologic	0.089	0.031	0.001	0.007
		Habitat	0.108	0.088	0.005	0.007
Yolk sac volume	0.235*	Hydrologic	0.315	0.948	–	–
Activity	0.121*	Habitat	0.215	0.415	–	–
Inter-individual distances	0.199*	Geographic	0.348	0.069	0.001	0.120
		Hydrologic	0.366	0.009	0	0.134
		Genetic	0.407	0.319	0.057	0.109
		Habitat	0.295	0.141	0.010	0.077

Analyses were performed between each KTA-based distance matrix (response variable) and all proxy-based (genetic distance, habitat divergence, geographic distance and hydrologic distance proxies) distance matrices. Some KTA are only correlated with a single proxy which remained after suppressors’ removal. With: Pearson’s correlation coefficient (r), β weights (β), and unique (U) and common (C) contributions of proxies to the variance of the response variable. (*) indicates significant R^2^ associated with a p-value < 0.05 (after Benjamini–Hochberg correction)

### Prioritisation procedure

A prioritisation procedure based on the sum of KTA-based or proxy-based distances was applied to our dataset. This procedure involved a k-mean clustering approach aiming at dividing all population pairs into k clusters (i.e. population pairs belonging to different clusters should be considered as different in terms of KTA or proxy; see Methods for details). The results obtained for the prioritisation strategy are presented in Table 2.

**Table 2:** Application of the prioritisation strategy

Population pair	Number of statistically significant KTA expression divergences/number of KTA investigated	Sum of KTA-based distances	KTA cluster identity	Standardised habitat divergence	Standardised hydrological distance	Standardised genetic distance	Sum of proxy-based distances	Proxy cluster identity
VAL-GEN	5/9	3.63	1	0.85	0.96	0.82	2.63	A
VAL-BOU	4/9	3.02	2	0.79	0.96	0.81	2.56	A
VAL-HOH	4/9	3.23	2	0.56	1	0.94	2.5	A
ISO-GEN	3/9	2.64	2	0.82	0.9	0.77	2.49	A
ISO-BOU	3/9	2.97	2	0.68	0.99	0.77	2.44	A
ISO-HOH	3/9	2.80	2	0.53	0.95	0.86	2.34	A
GEN-BAL	5/9	3.99	1	0.89	0.49	0.93	2.31	A
VAL-BAL	5/9	3.20	2	0.57	0.71	1	2.28	A
BOU-BAL	5/9	3.80	1	0.78	0.57	0.92	2.27	A
ISO-BAL	3/9	2.46	3	0.48	0.66	0.95	2.09	B
BOU-HOH	2/9	2.34	3	0.85	0.67	0.54	2.06	B
GEN-HOH	2/9	2.57	3	1	0.58	0.45	2.03	B
HOH-BAL	4/9	3.85	1	0.41	0.56	0.98	1.95	B
VAL-KIE	3/9	3.22	2	0.26	0.7	0.96	1.92	B
KIE-BAL	5/9	3.81	1	0.39	0.43	0.99	1.81	B
KIE-BOU	1/9	1.80	3	0.65	0.73	0.41	1.79	B
ISO-KIE	5/9	3.66	1	0.24	0.65	0.85	1.74	B
KIE-GEN	2/9	2.93	2	0.75	0.64	0.19	1.58	B
KIE-HOH	3/9	2.11	3	0.29	0.68	0.6	1.57	B
VAL-ISO	3/9	2.07	3	0.09	0.24	0.56	0.89	C
GEN-BOU	1/9	2.17	3	0.49	0.2	0	0.69	C

The number of KTA statistically different relatively to the total number of KTA is indicated for each population pair (according to Additional file 1: Fig. S1). The sum of KTA-based distances and proxy-based distances are provided for each population pair. The cluster identity is provided for KTA-based distances and proxy-based distances. Populations : lake Valkea-Müstajärvi (VAL), lake Iso-Valkjärvi (ISO), lake Kierzlinskie (KIE), lake Geneva (GEN), lake Bourget (BOU), lake Hohen Sprenzer (HOH), and lake Balaton (BAL)

When looking at proxies independently, the highest proxy-distance value corresponds to different population pairs depending on the proxy considered (Table 2). The prioritisation procedure indicates three clusters with the A cluster grouping populations presenting the highest values for the sum of proxy-based distances (Table 2). Populations belonging to cluster A mostly belong to clusters 1 and 2 when considering the sum of KTA-based distances and present high numbers of KTA statistically differentiated (Table 2).

## Discussion

### Assessment of KTA expression differentiation

Because we used a common garden experiment, we assumed that observed differentiations in the KTA expression have a genetic basis and are likely not due to phenotypic plasticity. Nevertheless, we used individuals sampled at the egg stage in the wild. Therefore, we cannot rule out that a part of KTA expression divergences is shaped by transgenerational effects or phenotypic plasticity [15, 51]. However, we argue that our experimental design has minimised as far as possible such potential biases. Another potential bias relies on the variable number of egg ribbons used between populations which could have influenced KTA results. However, we minimised the female specificity bias (i.e. maternal effects) by taking a large number of egg ribbons for each population.

### Single proxy approach: no silver bullet

We showed that no single proxy-based distance matrix is correlated with all KTA-based distance matrices. We found four significant correlations for genetic distance and two significant correlations for hydrologic distance and habitat divergence when we compared them with KTA-based distance matrices (Table 1). We also observed that each KTA is only significantly correlated with one proxy (Table 1).

Since inter-populational divergences can be shaped by (i) gene flow disruption/limitation and/or (ii) local adaptation to specific selective pressures, we were expecting that the assessments based on alternative proxies could lead to different results. Indeed, relationships between these two divergence-triggering factors and the alternative proxy-based distance matrices are different.

Although larger spatial disjunction, using geographic or hydrologic distance, increases the likelihood to reinforce these divergence-triggering factors (e.g. [52]), inter-populational differentiation can happen between adjacent populations, for instance due to a strong ecological barrier (e.g. for *Perca fluviatilis* [53]) or behavioural processes (e.g. natal homing behaviour [40]). Detections of such differentiations at small geographic scale are thus unlikely with spatial disjunction a priori (e.g. divergent results between hydrologic and survival rate distance with ISO, KIE, and BAL, see https://figshare.com/s/1067e47637c5572caaf7).

The habitat divergence proxy can theoretically overcome limitations of spatial disjunction proxies because it could highlight specific selective pressures at any geographic scale and, thus, inter-populational divergences (e.g. [54, 55]). Nevertheless, we observed only few significant correlations between KTA and habitat divergence. This can be explained because the habitat divergence proxy assessment most likely fails to highlight populations undergoing different selective pressures. First, the selection of relevant variables involved in local selective pressure is still hard to achieve. Indeed, the importance of environmental variables on population evolution is species-specific (and unsettled for *P. fluviatilis*) and cannot be known without long and difficult bioassays (e.g. [56]). Second, relevant environmental data on freshwater ecosystems are unevenly available for the different regions of the world. For instance, in our study, the worldwide databases of freshwater environmental variables (e.g. [57]) do not cover the whole studied sampling area. This led us to use terrestrial data in order to extrapolate aquatic environment characteristics (see similar strategy in [58, 59]), although it could potentially blur studied lake specificities. Optimally, analyses should be performed using data specific to lakes (e.g. lake physico-chemical parameters, feeding base) to improve the use of this proxy but the unavailability of sufficient data limits this strategy. Beside these difficulties, the habitat divergence proxy assessment can fail because extensive gene flow from adjacent regions can limit local adaptations [40] and, thus, minimise KTA expression divergences, even if specific environmental pressure occurs locally.

One could expect that the genetic distance proxy would be the most efficient to highlight populations with divergent KTA expressions. Indeed, it could detect populations with genetic divergences due to gene flow limitation/disruption triggered by spatial/hydrologic distance (i.e. isolation by distance), geographic barriers (i.e. geographic isolation), ecological barriers, or behavioural specificity (e.g. natal homing behaviour). However, our results highlight that not all KTA are correlated with the genetic distance proxy. Moreover, some traits which are not correlated with the genetic distance proxy are correlated with another proxy. To explain the absence of correlation of some KTA with the genetic distance proxy, it could be hypothesised that populations could have diverged recently, implying that few divergences in neutral markers had time to appear [60, 61].

### How to better catch the divergence: a multi-proxy strategy

Because there is no proxy-based distance correlated with all KTA-based distances, highlighting populations that likely display KTA expression divergences should be based on the use of several proxies. Genetic distance and habitat divergence proxies are theoretically complementary to maximise the detection of such populations. On the one hand, the genetic distance proxy is relevant to highlight divergence by distance or populations with divergent demographic histories while the habitat divergence proxy can reflect local adaptations which are not (already) visible in neutral markers. On the other hand, populations occurring in similar habitats can display phenotypic divergences, including in KTA. Thus, the use of these two proxies allows taking into consideration both gene flow disruption/limitation and local adaptation to specific selective pressures. However, these proxies can be sometimes misleading due to their intrinsic limitations (see before), particularly the habitat divergence proxy (unless performing the analysis with more relevant lake variables). One way to mitigate their limitations relies on the combination of these two proxies with the hydrologic distance proxy (according to our results, the geographic distance proxy can be beneficially replaced by hydrologic distance proxy, which is more relevant for aquatic species and is the only one correlated to some KTA-based distance matrices). The hydrologic distance proxy could be correlated with both phenotypic differentiation by distance and local adaptations between distant populations. Moreover, the hydrologic distance proxy is correlated in our study with two KTA which are not correlated neither with genetic distance or habitat divergence proxies. Even though its calculation might be time and system memory-consuming, it requires no specific information except for geographic coordinates which makes it simple and useable in all scenarios. Here, we promote the use of a multi-proxy approach to pinpoint a population or population group to be assessed for KTA expression divergences. Even if some proxies appear as less efficient, their simple evaluation requiring little information (e.g. geographic distance from GPS coordinates) makes their use valuable to increase chances of finding out divergences in KTA. Another potential alternative would rely on ranking the KTA since not all KTA have the same importance to stakeholders. This strategy would consist of choosing the proxy which is correlated to the most important KTA (e.g. growth rate and survival rate [45]). However, this strategy is not optimal since a successful domestication process requires the favourable expression of several key traits involved in various biological functions.

Regarding the multi-proxy strategy, it is likely that the different proxies drive to divergent conclusions regarding which populations should be considered. This implies integrating all proxies in the same decision framework to highlight population pairs that should be considered in common garden experiments. Recommending for such experiments any population pair that is somewhat divergent for one or more proxies would fail to limit the number of populations involved in costly bioassays. Therefore, prioritisation procedures should be developed.

### An example of prioritisation procedure exemplified with our test-case species

We recommend evaluating population/population group pairs according to both their degree of divergences in each proxy and the degree of congruence in divergence of all proxies. We applied here a prioritisation procedure on our dataset to validate this procedure.

Firstly, in this example, depending on the proxy considered, the conclusion regarding which population pairs are the most interesting would differ (Table 2). For instance, considering the genetic proxy, the highest distance is seen between VAL and BAL while with hydrologic and habitat proxies, the comparisons VAL-HOH and GEN-HOH would be promoted, respectively. This confirms the need for a multi-proxy approach and a prioritisation procedure to make a consensus between proxies. Second, the k-mean clustering approach allows to highlight groups of population pairs for which sum values can be considered as similar. The highest proxy-based distance sum values were shown for cluster A. When comparing to KTA-based clustering, its groups population pairs belonging to clusters 1 and 2, except for five population pairs, and exclude all population pairs belonging to cluster 3. Population pairs belonging to cluster A also present a high number of KTA significantly differentiated. The match between clusters obtained with KTA-based distances and proxy-based distances is not perfect but this is expected since (i) not all KTA are correlated to each proxy and (ii) the correlation value is inferior to 1. Overall, this procedure allows to limit the number of population pairs, here to the nine population pairs belonging to cluster A. Any of those nine population pairs could be considered for further bioassays and would be likely to present divergences in KTA. The final number of pairs that will be effectively evaluated in bioassays will also depend on other pragmatic and economic factors which will further decrease the final number of pairs considered. Those factors include, among others, biological sampling possibilities and limitations (e.g. TRACES rules), distance from the aquaculture facilities which impacts sampling costs, availability of experimental facilities, as well as financial and human means.

## Conclusion

Our results show differences in the correlation degree between the alternative proxy-based and key trait-based distance matrices. Although we show that the genetic proxy is the most correlated to phenotypic key traits, no proxy was correlated to all inter-populational divergences in key traits. Therefore, we argue that the use of a multi-proxy approach is the most efficient and a population prioritisation strategy is proposed to optimize it.

We obtained this conclusion by performing a retrospective study (i.e. after having performed bioassays) and *P. fluviatilis* populations of interest have been highlighted in previous studies [33, 44]. For this species, the population prioritisation might be mainly useful to integrate wild specimens from highlighted populations to improve current fish stocks since European perch is already produced. However, compared to other well-known aquacultural fish species, the domestication process is quite recent in *P. fluviatilis* [62] and this study could therefore optimise the choice of the founder populations in new fish farms.

We argue that the multi-proxy approach can be applied on new candidate species for aquaculture for which KTA information are completely unknow. Indeed, it constitutes a first step, prior to domestication, that allows identifying population pairs which are likely differentiated in KTA and which are worth to be considered for bioassays. This will limit the trial-and-error approach and the random selection of populations which are further assessed in bioassays. Moreover, the calculation of those proxies will also provide additional information such as genetic diversity/variability, which are useful in the following domestication steps to avoid inbreeding issues and estimate potential for selective breeding programs [7]. Overall, the use of the multi-proxy approach will be useful to help further aquaculture development with other fish species. However, it still requires to be validated on other test-case species prior to large-scale application to aquaculture.

## Methods

### Test case species

The European perch is a freshwater species which is widespread across a diverse range of habitats in Eurasia [63]. Due to its high socio-economic value (i.e. food market, recreational interest), this species started to be produced in the 1990’s, mostly in intensive recirculated aquaculture systems (RAS) [62, 64]. A geographic differentiation was already shown for *P. fluviatilis* in standardised conditions for KTA related to growth (e.g. [65, 66]), development (e.g. [33, 67]), and behaviour (e.g. [44, 49]). This makes the species an appropriate test-case to assess the relevance of alternative proxies to highlight KTA differentiation among wild populations.

### KTA evaluation in aquaculture standardised conditions

All procedures used in this study were in accordance with national and international guidelines for protection of animal welfare (Directive 2010/63/EU). This study was conducted with the approval Animal Care Committee of Lorraine (CELMA n°66) and the French Ministry of Higher Education, Research, and Innovation (APAFIS13368-2018020511226118, APAFIS17164-2018101812118180).

**Fig. 1:**
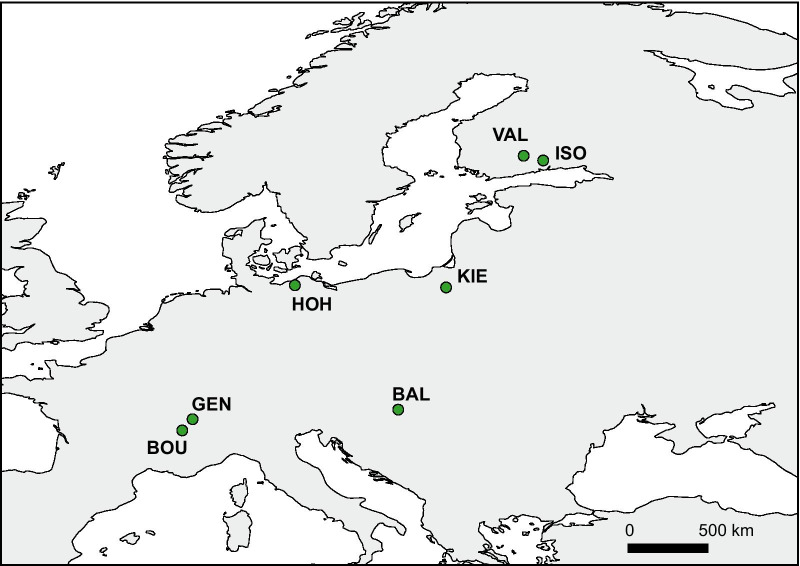
Map representing the seven wild *Perca fluviatilis* populations sampled. *VAL* Valkea-Müstajärvi, *ISO* Iso-Valkjärvi, *KIE* Kierzlinskie, *GEN* Geneva, *BOU* Bourget, *HOH* Hohen Sprenzer, and *BAL* Balaton

Egg ribbons were collected during the spawning seasons across two years (May 2018 and April–May 2019). Seven lakes were sampled (Fig. 1): Valkea-Müstajärvi (VAL; 2018, Finland; WGS84: 61° 13′ 08″ N, 25° 07′ 05″ E), Iso-Valkjärvi (ISO; 2018, Finland; WGS84: 60° 57′ 21″ N, 26° 13′ 3″ E), Kierzlinskie (KIE; 2019, Poland; WGS84: 53° 47′ 54″ N, 20° 44′ 45″ E), Geneva (GEN; 2019, France; WGS84: 46° 22′ 7.20″ N, 6° 27′ 14.73″ E), Bourget (BOU; 2019, France; WGS84: 45° 44′ 12.469″ N, 5° 52′ 1.617″ E), Hohen Sprenzer (HOH; 2019, Germany; WGS84: 53° 55′ 10.369″ N, 12° 13′ 6.005″ E), and Balaton (BAL; 2019, Hungary; WGS84: 46° 54′ 23.375″ N, 18° 2′ 43.119″ E). After transportation, 13 to 32 ribbons per lake were incubated at 13 °C at 400 lx (for incubation details, see [33, 49]).

The experiment, performed in a RAS, started at one day post-hatching (dph) until the end of weaning, at 26 dph. For each population, after hatching, larvae from the different egg ribbons were mixed and transferred to three green internal-wall cylindro-conical tanks (three replicates per population) at a density of 50 larvae/L at our experimental platform of aquaculture (Unit of Animal Research and Functionality of Animal Products, University of Lorraine, Vandœuvre-lès-Nancy, France). Temperature was gradually raised to 20 °C (1 °C rise per day). Photoperiod was 12L:12D and light intensity stayed constant during the lighting period at 400 lx at the water surface (with simulation of dawn and dusk for 30 min). Larvae were hand-fed from 3 days post-hatching, seven times a day, during the illuminated period, every 1h30, with newly hatched *Artemia* nauplii (Sep-Art, INVE). At 16 dph, weaning started: *Artemia* ration was diminished by 25% every three days while ration of dry feed [BioMar (Nersac, France), 300 µm until 21 dph, then 500 µm] was increased in the same proportions. After 25 dph, larvae were only fed with dry feed. Tanks were cleaned daily after first-feeding and dead larvae were removed and counted. Oxygen and temperature were checked daily while nitrite and ammonium concentrations and pH were monitored three times a week. At the end of the experiment, larvae left in each tank were counted and sorted according to the presence/absence of swim bladder inflation (following protocol used in [68]) and skeletal deformities. Additional experimental details, including water parameters values, are provided in Toomey et al. [33].

Survival rate was calculated using the following formula: Nf*100/(Ni − Ns), in which Nf is the final number of larvae counted at the end of experiment, Ni the initial number of individuals, and Ns the number of larvae sampled along the experiment (i.e. sampling for behaviour experiments, see below). In order to evaluate growth traits, 30 larvae per population (i.e. ten larvae per cylindro-conical) were sampled the first and last days of the experiment. After sampling, larvae were euthanized with an overdose of MS-222 and preserved in formalin 4%. Individuals were measured for total length in ImageJ [69] (± 0.01 mm). Specific growth rate (SGR) was calculated using the following formula: SGR = 100*(ln(Lf) − ln(Li))*∆T^−1^ where Li and Lf are respectively the initial and final length and ∆T the length of experiment. Final growth heterogeneity was calculated in the following way: CV_Lf_/CV_Li_ where CV is the coefficient of variation (100*standard deviation/mean) and Li and Lf the initial and final length, respectively. Swim bladder inflation rate was calculated in the following way: 100*(SB + /Nf) with SB + the number of larvae with swim bladder and Nf the final number of larvae. Deformity rate was evaluated using the following formula: 100*(Nm/Nf) with Nm the number of deformed larvae (visible skeletal deformities) and Nf the final number of larvae. Aggressiveness, including enucleation which is a specific aggressive behaviour in *P. fluviatilis* [70], was evaluated based on the daily examination of dead larvae using the following formula: (Ne + Nt)/Nd where Ne is the number of enucleated larvae (i.e. missing one eye), Nt the number of truncated larvae (cannibalism type I; [71]), and Nd the number of dead larvae counted between five and 26 dph (not possible to count dead larvae the first five days but aggressive interactions are reported to start at later stages [45, 72]).

The protocol to evaluate inter-individual distances [73] and activity is adapted from [49, 74]. All methods were performed in accordance with the relevant guidelines and regulations. In a nutshell, each population was evaluated at 25 and 26 dph. For each population, 90 larvae (i.e. 30 larvae per cylindro-conical tank) were sampled the day before the experiment and transferred to aquaria (58 L; 80 lx) at 20 °C. After one night of acclimatisation, populations were tested by groups of ten larvae which were placed in circular arenas (10 lx, 30 cm diameter, 1.5 cm of water depth) and filmed (three arenas tested simultaneously; three replicates per cylindro-conical tank, nine replicates per population). After 30 min acclimatisation, the following 30 min were used to evaluate activity and inter-individual distances. At the end of the experiment, larvae were euthanized with an overdose of MS-222 for further length measurements. Mean total lengths of larvae tested from VAL, ISO, KIE, GEN, BOU, HOH, and BAL were respectively 12.90 ± 0.62 mm, 14.05 ± 0.55 mm, 10.90 ± 0.73 mm, 11.81 ± 1.01 mm, 11.77 ± 0.48 mm, 11.35 ± 0.72 mm, and 10.62 ± 0.47 mm. Images were extracted from videos every five minutes (six images per video) to evaluated inter-individual distances (i.e. mean of distances between a given individual and all the other individuals of the group; group cohesion indicator [73]). Analyses were made in ImageJ [69]. For each image, coordinates of each individual (using the middle point between the eyes) were noted. This allowed measuring distances between a given individual and the other individuals of the group and all these distances were averaged to obtain one value per individual. The mean of values of all group members were averaged per image. Finally, and the mean between all image values allowed getting a mean value of inter-individual distances per replicate [74]. Regarding activity, one image per second was extracted for six consecutive seconds every five minutes. Distance swam every second for five seconds was calculated and the mean allowed obtaining the distance swam per second for each individual. The mean between individual values allowed getting an activity for each image series. The mean between image series allowed calculating the activity per replicate.

### Distance matrix for the different proxies

In the present study, we did not use any phenotypic proxy because morphological and/or behavioural information was not available for all wild perch populations. Moreover, we did not estimate such a proxy on larvae reared in the bioassays because these phenotypic traits can be strongly influenced by the environment (e.g. [75]). Therefore, we only evaluated three proxies: (i) genetic distance, (ii) habitat divergence, and (iii) geographic/hydrologic distance.

#### Genetic distance proxy

The genetic assessment of the seven populations was performed on 10 individuals per population collected randomly at the end of the experiment. Larvae were stored in 99% ethanol at − 20 °C until analyses. Samples were sent to Genoscreen (Lille, France) for DNA isolation, marker amplification, and Sanger sequencing. Three mitochondrial regions were studied (D-loop of control region, cytochrome b, and 16S rRNA; see references for primers in [76]) following the protocol available in Toomey et al. [76]. Both strands of each PCR product were sequenced. Consensus sequences of mitochondrial regions were computed and edited using CodonCode Aligner 7.1.2 (CodonCode Corporation, Dedham, Massachusetts, USA). There was no uncertainty in the consensus sequences. The *P. fluviatilis* origin of each sequence was verified using BLAST [77]. Sequence alignment was performed in MAFFT (default parameters; [78]). Translation to proteins for Cytb was performed in Mesquite 3.20 [79]. A tandemly repeated array was identified in D-loop (previously reported in previous *P. fluviatilis* studies; [80]) and mutations in the repeated array were coded as a single mutational step for further analyses. Mitochondrial markers were concatenated within a single alignment for further analyses using Mesquite. Haplotype sequences were deposited in GenBank (GenBank accession numbers: MN939382 to MN939395). Genetic distances between populations were calculated in R using ɸ_ST_ distance [81] in SPADS [82].

#### Habitat divergence proxy

We used 19 bioclimatic data (BIO1 to BIO19; https://www.worldclim.org/data/bioclim.html) as well as annual mean solar radiation (kJ m^−2^ day^−1^), annual mean wind speed (m s^−1^), and annual mean water vapor pressure (kPa) from WorldClim Version2 to assess sampling place environments. We considered these abiotic environmental parameters (at the sampling location) recorded between 1970 and 2000 at a resolution of 10 arc‐minutes [83]. We also used four additional variables: lake area (km^2^), catchment area (km^2^), average and maximum depths (m), and altitude (m). All variables were standardised to zero mean and unit variance due to their different scales of measurement (package “vegan” [84]). A principal component analysis was performed (Additional file 3: Table S1; packages “MASS” [85] and “factoextra” [86]) using the Kaiser–Guttman rule to determine the number of axes to retain (here the first four axes representing 98.0% of the variance). Coordinates for each population replicate were extracted from the first four axes and a distance matrix using the Bray–Curtis distance method was calculated (R package “vegan”).

#### Geographic and hydrologic distance proxies

Geographic distance matrix was set through the calculation of the great-circle distances (i.e. shortest distance over the earth’s surface) according to the “Vincenty” (ellipsoid) method [87] implemented in the R package “geosphere” [88]. Because this geographic distance is a priori not relevant for fish species, we also estimated a hydrologic distance using an algorithm based on circuit theory and implemented in the program Circuitscape [89]. Rivers and lakes shapefiles were respectively extracted from the Catchment Characterisation and Modelling river and catchment database v2 [90] and HydroSHEDS [91] and from Natural Earth (www.naturalearthdata.com) databases, and jointly rasterised on a geo-referenced grid with a resolution of 0.5 arcmin. The algorithm implemented in Circuitscape computes pairwise electric resistances by treating a raster as a grid of electric resistance or conductance. In the present case, we used Circuitscape to compute hydrologic distances measured as electric resistance between locations when treating the rivers raster as a grid of electric conductance. One of the advantages of the Circuitscape algorithm is that it considers the contribution of several potential pathways to compute pairwise distances (resistances) between locations. Specifically, we assigned a value of 1 to terrestrial raster cells that were not crossed by a river or occupied by a lake and a value of 1 + *k* for terrestrial raster cells crossed by a river or occupied by a lake, the parameter *k* thus defining to what extent raster cells crossed by a river are more conductible (i.e. facilitate movement) than raster cells that are not crossed by a river. To explore the impact of *k* on the outcome of our analyses, we tested three different values for *k*: *k* = 10, 100, and 1000. These three different values led to highly similar results and did not alter our conclusions (results not shown). Therefore, we only report results based on hydrologic distances computed with *k* = 100.

### Relevance of the different proxies

In order to create a trait-by-trait distance matrix, we first checked the statistical differentiation (p-value < 0.05) of phenotypic traits between populations: we tested normality of distribution and homogeneity of variances using a Shapiro–Wilk test and a Levene test, respectively (R package “lawstat” [92]). When assumptions were not respected, data were log-transformed. To check the influence of the cylindro-conical tank on results, we compared linear (phenotypic traits as fixed factors, no random factor) and linear mixed models (cylindro-conical tanks as random factor and phenotypic traits as fixed factors; R package “lmer” [93]) using the corrected Akaike Information Criterion (AICc; R package “qpcR” [94]. For most traits, there was no significant influence of the cylindro-conical tank on the model. Therefore, we used one-way analyses of variance (ANOVA F-test), followed by Tukey post hoc tests (R package “stats”), to evaluate differences between populations. When the effect of the cylindro-conical tank was significant, we performed the ANOVA on the linear mixed model and estimated marginal means were calculated (R package “emmeans” [95]. Regarding aggressive rate and activity, assumptions were not met despite log-transformation. Thus, Kruskal–Wallis H tests and Dunn post hoc test (R package “PMCMR” [96] were used. Traits presenting no significant differentiation between populations were excluded from further analyses.

A trait-by-trait distance matrix was created using the Euclidean method (R package “vegan”, three replicates per population). All distance matrices are available in Figshare (https://figshare.com/s/1067e47637c5572caaf7). We performed multivariate analyses using multi-regression on distance matrices (1000 permutations) coupled with commonality analyses (MRDM-CA) using R packages “ecodist” [97] and “yhat” [98]. For these analyses, all proxy-based distance matrices were preliminary standardised. The multi-regression on distance matrices (MRDM) analysis allows estimating the Pearson correlation coefficient r (i.e. direct effect of the proxy on the response variable, irrespectively from the influence of other proxies) and beta weights (i.e. total effect of the proxy on the response variable, considering the contribution of other proxies). The commonality analysis (CA) is a detailed variance-partitioning procedure which allows taking into consideration collinearity between proxies [99]. This analysis estimates the unique (“U”; amount of variance in the KTA accounted for by each single proxy) and common (“C”; variance jointly explained by several proxies) contributions of each proxy. After the first round of MRDM-CA, total suppressors were identified and discarded because they can be responsible for artefactual relationships among variables. This can allow purifying the relationships between the remaining proxies and the response variable (i.e. in our case the KTA-based distance matrices) [99]. Successive MRDM-CA were performed until all suppressors were removed. A proxy was considered as a total suppressor when unique contribution is counter-balanced by its (negative) common contribution (classical suppression) and/or when regression and correlation coefficients are of opposite signs (cross-over suppression; [99, 100]). In addition, we investigated correlations between trait-based and proxy-based distance matrices using Mantel tests in R (R package “vegan”; 9,999 permutations). Four Mantel tests were performed for each KTA: (i) ɸ_ST_ genetic distance vs KTA-based distance matrix, (ii) habitat divergence vs KTA-based distance matrix, (iii) geographic distance vs KTA-based distance matrix, and (iv) hydrologic distance vs KTA-based distance matrix.

### Prioritisation procedure

We here propose a prioritisation procedure based on our dataset. First, all KTA-distance and proxy-distance matrices were standardised between 0 and 1 (all raw proxy and KTA matrices are available at https://figshare.com/s/1067e47637c5572caaf7). Second, the average distance value among replicates was calculated for each proxy/KTA of each population pair. Third, the sum of the three proxy-distance values was calculated per population pair. Similarly, the sum of all KTA-distance values was also calculated per population pair. Relevant ranking of population pairs according to the sum of KTA or proxy values must take into account that pairs with similar sum values should be regarded as equivalent and no prioritisation among them cannot be proposed (i.e. indifference between pairs). Therefore, we chose to use a k-means clustering procedure to determine indifference groups of population pairs. It aims at partitioning population pairs into k clusters in which each population belongs to the cluster with the nearest centroid. For each sum variable (i.e. sum of KTA distances and sum of proxy distances), the optimal number of clusters (k) was determined using the Elbow method (R package “factoextra” [86]). Then, a k-means clustering was performed for dividing all population pairs into k clusters for each sum variable independently (R package “stats”).

## Supplementary Information

**Additional file 1: Figure S1.** Principal component analysis biplot representing environmental variables (in blue) and populations (dots in grey) using the first two axes. Populations: BAL: Balaton, VAL : Valkea-Müstajärvi, ISO: Iso-Valkjärvi, KIE: Kierzlinskie, GEN: Geneva, BOU: Bourget, HOH: Hohen Sprenzer. BIO1 to BIO19 correspond to the bioclimatic variables from Worldclim. Vap, wind, and srad correspond to water vapor pressure, wind speed, and solar radiation, respectively.

**Additional file 2: Figure S2.** Barplots representing results obtained for all key traits studied (n = 3 per population, except for activity and inter-individual distances for which n=9). Different letters indicate significant differences between populations (p-value<0.05) using post-hoc tests.

**Additional file 3: Table S1.** Correlation results between proxy-based (geographic, hydrologic, genetic, and habitat proxies) and KTA-based distance matrices with r the Mantel correlation value and its associated p-value (significant r (p-value<0.05) are indicated in bold).

## Data Availability

The datasets supporting the conclusions of this article are available in the Figshare (https://doi.org/10.6084/m9.figshare.11407128 / https://figshare.com/s/1067e47637c5572caaf7) for proxy-based matrices and in Genbank (MN939382 to MN939395) for haplotype sequences.

## Abbreviations

AICc: Corrected Akaike Information Criterion
BAL: Lake Balaton
BOU: Lake Bourget
C: Common contribution
CA: Commonality analysis
GEN: Lake Geneva
ISO: Lake Iso-Valkjärvi
KIE: Lake Kierzlinskie
MRDM: Multi-Regression on Distance Matrices
RAS: Recirculation Aquaculture System
SGR: Specific growth rate
KTA: Key traits for aquaculture
U: Unique contribution
VAL: Lake Valkea-Müstajärvi
