# Supplementary material for: Split it up and see: using proxies to highlight divergent inter-populational performances in aquaculture standardised conditions
Source: BMC Ecol Evol. 2021 Nov 22;21:206. doi: 10.1186/s12862-021-01937-z (PMC8607704; doi:10.1186/s12862-021-01937-z)

Figure S1: Principal component analysis biplot representing environmental variables (in blue) and populations (dots in grey) using the first two axes. Populations: BAL: Balaton, VAL : Valkea-Müstajärvi, ISO: Iso-Valkjärvi, KIE: Kierzlinskie, GEN: Geneva, BOU: Bourget, HOH: Hohen Sprenzer. BIO1 to BIO19 correspond to the bioclimatic variables from Worldclim. Vap, wind, and srad correspond to water vapor pressure, wind speed, and solar radiation, respectively.

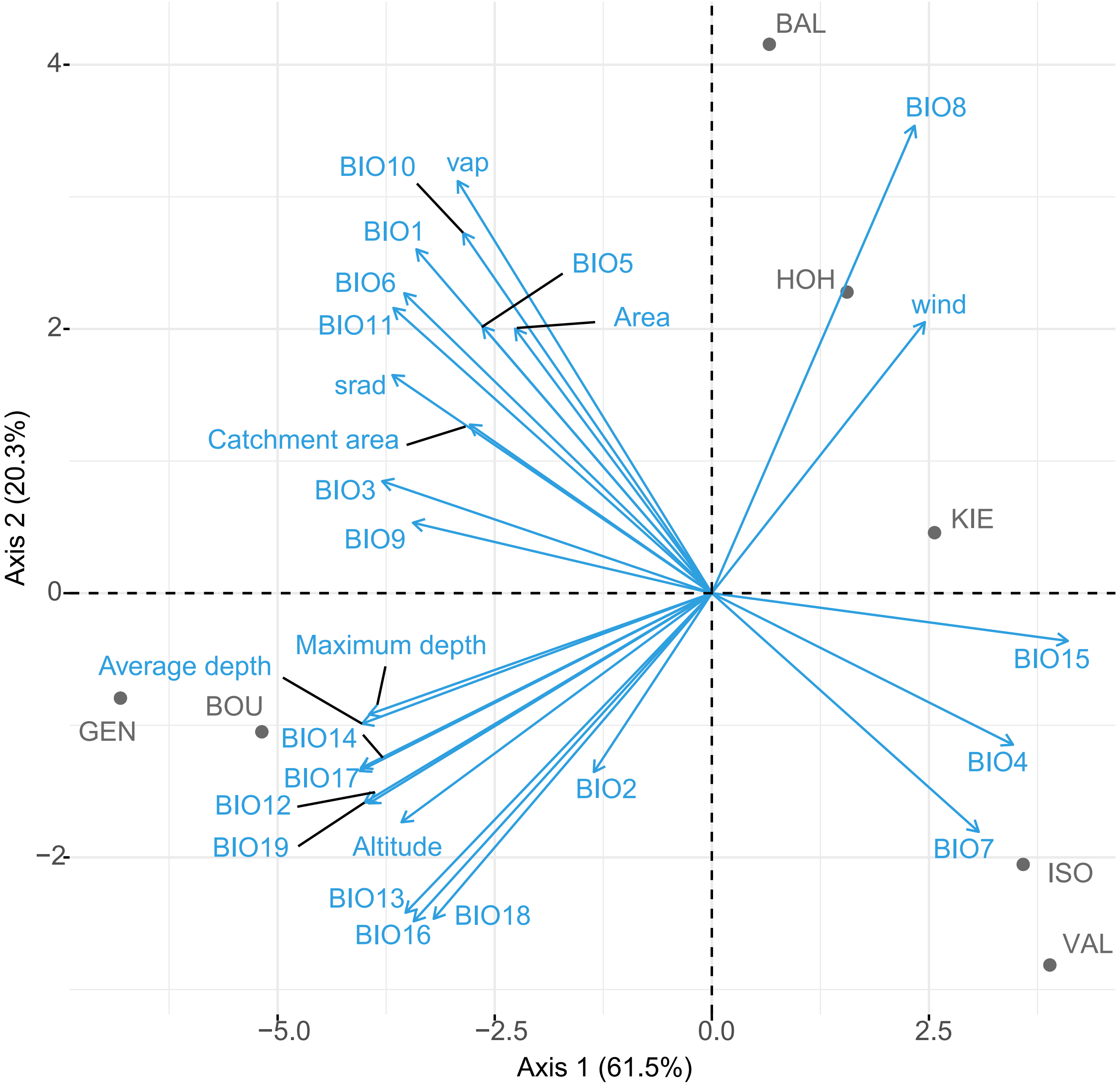

Supplement: Supplementary file 1 — Additional file 1: Figure S1. Principal component analysis biplot representing environmental variables (in blue) and populations (dots in grey) using the first two axes. Populations: BAL: Balaton, VAL : Valkea-Müstajärvi, ISO: Iso-Valkjärvi, KIE: Kierzlinskie, GEN: Geneva, BOU: Bourget, HOH: Hohen Sprenzer. BIO1 to BIO19 correspond to the bioclimatic variables from Worldclim. Vap, wind, and srad correspond to water vapor pressure, wind speed, and solar radiation, respectively. [file 12862_2021_1937_MOESM1_ESM.pdf]
