# Supplementary material for: Split it up and see: using proxies to highlight divergent inter-populational performances in aquaculture standardised conditions
Source: BMC Ecol Evol. 2021 Nov 22;21:206. doi: 10.1186/s12862-021-01937-z (PMC8607704; doi:10.1186/s12862-021-01937-z)

Figure S2: Barplots representing results obtained for all key traits studied (n = 3 per population, except for activity and inter-individual distances for which n=9). Different letters indicate significant differences between populations (p-value<0.05) using post-hoc tests.

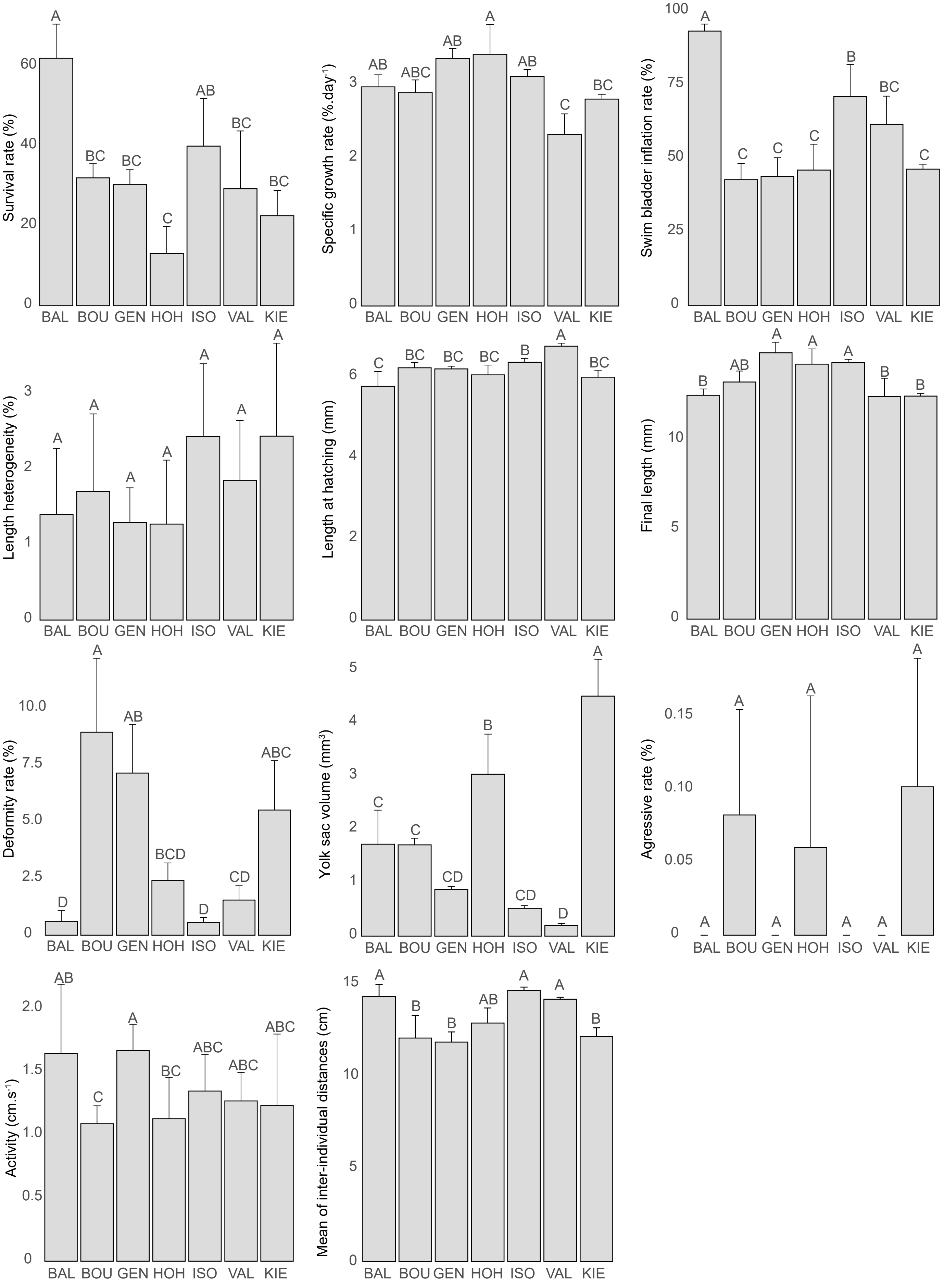

Supplement: Supplementary file 2 — Additional file 2: Figure S2. Barplots representing results obtained for all key traits studied (n = 3 per population, except for activity and inter-individual distances for which n=9). Different letters indicate significant differences between populations (p-value<0.05) using post-hoc tests. [file 12862_2021_1937_MOESM2_ESM.pdf]
