# Supplementary material for: Split it up and see: using proxies to highlight divergent inter-populational performances in aquaculture standardised conditions
Source: BMC Ecol Evol. 2021 Nov 22;21:206. doi: 10.1186/s12862-021-01937-z (PMC8607704; doi:10.1186/s12862-021-01937-z)
Supplement: Supplementary file 3 — Additional file 3: Table S1. Correlation results between proxy-based (geographic, hydrologic, genetic, and habitat proxies) and KTA-based distance matrices with r the Mantel correlation value and its associated p-value (significant r (p-value<0.05) are indicated in bold). [file 12862_2021_1937_MOESM3_ESM.pdf]

Table S1: Correlation results between proxy-based (geographic, hydrologic, genetic, and habitat proxies) and KTA-based distance matrices with r the Mantel correlation value and its associated p value (significant r (p-value<0.05) are indicated in bold).

KTA	Mantel test (r) results			
	Geographic distance proxy	Hydrologic distance proxy	Genetic distance proxy	Habitat divergence proxy
Survival rate	r=0.035 (p-value=0.260)	r=0.042 (p-value=0.222)	**r=0.505** (p-value= $1x10^{-4}$)	r=-0.082 (p-value=0.183)
Swim bladder inflation rate	**r= 0.199** (p-value=0.017)	**r=0.142** (p-value=0.037)	**r=0.631** (p-value= $1x10^{-4}$)	**r=0.182** (p-value=0.043)
Deformity rate	**r=0.350** (p-value=0.001)	**r=0.286** (p-value=0.001)	**r=0.193** (p-value=0.034)	**r=0.469** (p-value=$6x10^{-4}$)
Specific growth rate	r=0.077 (p-value=0.114)	**r=0.237** (p-value=0.002)	r=0.133 (p-value=0.110)	r=0.070 (p-value=0.237)
Initial length	**r=0.223** (p-value=0.004)	**r=0.244** (p-value=0.001)	**r=0.472** (p-value=$1x10^{-4}$)	r=0.020 (p-value=0.399)
Final length	r=0.038 (p-value=0.212)	r=0.086 (p-value=0.102)	r=0.039 (p-value=0.294)	r=0.108 (p-value=0.110)
Yolk sac volume	r=0.099 (p-value=0.075)	**r=0.315** (p-value=0.001)	**r=0.217** (p-value=0.024)	r=0.005 (p-value=0.424)
Activity	r=0.034 (p-value=0.255)	r=-0.053 (p-value=0.792)	r=0.016 (p-value=0.381)	**r=0.215** (p-value=0.028)
Inter-individual distances	**r=0.348** (p-value=0.002)	**r=0.366** (p-value=$5x10^{-4}$)	**r=0.407** (p-value=$1x10^{-4}$)	**r=0.295** (p-value=0.003)
